# Chromosome-level genome assembly and annotation of the Antarctica whitefin plunderfish *Pogonophryne albipinna*

**DOI:** 10.1038/s41597-023-02811-x

**Published:** 2023-12-12

**Authors:** Euna Jo, Soyun Choi, Seung Jae Lee, Jinmu Kim, Eun Kyung Choi, Minjoo Cho, Jangyeon Kim, Sangdeok Chung, Jaebong Lee, Jeong-Hoon Kim, Hyun Park

**Affiliations:** 1 Department of Biotechnology, College of Life Sciences and Biotechnology, Korea University, Seoul, 02841 Korea; 2 Division of Life Sciences, Korea Polar Research Institute (KOPRI), Incheon, 21990 Korea; 3 National Institute of Fisheries Science (NIFS), Busan, 46083 Korea

**Keywords:** Genome evolution, Eukaryote

## Abstract

The Antarctic whitefin plunderfish *Pogonophryne albipinna* belongs to the family Artedidraconidae, a key component of Antarctic benthic ecosystems within the order Perciformes and the suborder Notothenioidei. While genome research on *P. albipinna* using short-read sequencing is available, high-quality genome assembly and annotation employing long-read sequencing have yet to be performed. This study presents a chromosome-scale genome assembly and annotation for *P. albipinna*, utilizing a combination of Illumina short-read, PacBio long-read, and Hi-C sequencing technologies. The resulting genome assembly spans approximately 1.07 Gb, with a longest scaffold measuring 59.39 Mb and an N50 length of 41.76 Mb. Of the 1,111 Hi-C scaffolds, 23 exceeded 10 Mb and were thus classified as chromosome-level. BUSCO completeness was assessed at 95.6%. The assembled genome comprises 50.68% repeat sequences, and a total of 31,128 protein-coding genes were predicted. This study will enhance our understanding of the genomic characteristics of cryonotothenioids and facilitate comparative analyses of their adaptation and evolution in extreme environments.

## Background & Summary

The Artedidraconidae family, part of the suborder Notothenioidei within the order Perciformes, plays a significant role in Antarctic benthic ecosystems. It accounts for a substantial portion of fish species diversity in the high Antarctic Zone, Weddell Sea, and Ross Sea^1–5^. Comprising four genera—*Artedidraco, Dolloidraco, Histiodraco*, and *Pogonophryne*—Artedidraconids feature a mental barbel with species-specific morphology^6–12^. Traditional taxonomy identifies 27 species within the genus *Pogonophryne*, the most diverse among Antarctic notothenioids^13^. However, recent research suggests that this species diversity may be overestimated^14,15^. Specifically, Parker *et al*.^14^ proposed condensing the majority of *Pogonophryne* species into five (or six, if new species are included) based on comprehensive analyses of phylogenomic data and morphological traits. Eastman and Eakin^15^ further organized the 27 *Pogonophryne* species into five groups within three categories: the *P. albipinna* group (unspotted), and the *P. barsukovi, P. marmorata, P. mentella* groups (dorsally spotted), as well as the *P. scotti* group (dorsally unspotted).

Among these, *P. albipinna*, also known as the whitefin plunderfish, is a representative species of the *P. albipinna* group. It is distinguished not only by a lack of dark spots on its head and trunk but also by its predominantly white fins and its habitat in water depths exceeding 1,500 meters^10,15–17^. Although genome studies on *P. albipinna* have been published, such as a complete mitochondrial genome report^18^ and a preliminary genome survey^19^, research employing state-of-the-art technologies for high-quality genome assembly and gene annotation has not been conducted. Furthermore, while the chromosome number for other *Pogonophryne* species, such as *P. barsukovi, P. marmorata, P. mentella*, and *P. scotti*, has been established through cytogenetic studies as 2n = 46^20,21^, the chromosome number for *P. albipinna* remains unidentified.

Recent research has focused on the genomic characteristics of Antarctic fish species, revealing whole genome sequence and assembly data. These studies also provide genomic insights into adaptations to low-temperature environments, including genes associated with freeze resistance, oxygen-binding, and oxidative stress^22–29^. The genus *Pogonophryne* is hypothesized to exhibit specific features for cold-water adaptation, such as functional alterations in hemoglobin or the presence of antifreeze glycoprotein (AFGP). For example, *P. favosa* possesses a specialized structure, convexitas superaxillaris, located beneath the base of the pectoral fin, which secretes antifreeze proteins^30^. In a separate study, the amino acid sequences and ligand-binding properties of hemoglobin were examined in two species of Artedidraconidae (*Artedidraco orianae* and *P. scotti*). These species demonstrated unexpectedly high oxygen affinity, contrasting with the hemoglobin deficiency observed in channichthyid icefish^31^.

In this study, we performed a chromosome-level genome assembly and annotation of *P. albipinna*, utilizing PacBio long-read sequencing and high-throughput chromosome conformation capture (Hi-C) technology. This work aims to elucidate the genomic characteristics of Antarctic fish and may serve as a basis for further investigations into their adaptation and evolutionary responses to extreme environments.

## Methods

### Sampling and DNA extraction

Samples of *P. albipinna* were collected from the Ross Sea, Antarctica (77°05′S, 170°30′E in CCAMLR Subarea 88.1) and subsequently transported to the Korea Polar Research Institute (KOPRI) in a frozen state. Muscle tissues were excised from these frozen specimens for the extraction of high molecular weight (HMW) DNA using a conventional phenol/chloroform-based method. Molecular identification of the species was carried out using a primer set (FishF2 and FishR2) specifically designed to amplify the mitochondrial cytochrome c oxidase I (COI) gene region^32^.

### Long-read sequencing and assembly

The extracted HMW DNA was utilized to construct 20 kb size-selected PacBio Sequel libraries, following the manufacturer’s protocol and employing the BluePippin size-selection system (Sage Science, Beverly, MA, USA). Specifically, the SMRTbell library was prepared using the SMRTbell Template Prep Kit 1.0, and the SMRTbell-polymerase complex was generated using the Sequel Binding Kit 3.0 (Pacific Biosciences, Menlo Park, CA, USA). This complex was then loaded into SMRT cells 1 M v3 and sequenced with the Sequel Sequencing Kit 3.0 (Pacific Biosciences, Menlo Park, CA, USA) for a 600-min movie time per cell. The genome of *P. albipinna* was sequenced using six PacBio SMRT cells, generating 7,776,779 raw reads with a total bases of approximately 81.11 Gb (Table 1). De novo genome assembly was performed using FALCON-Unzip assembler v0.4^33^, with parameter settings of length_cutoff = 12,000 and length_cutoff_pr = 10,000. Subsequently, the draft genome assembly was polished using Pilon v1.23^34^ to enhance its accuracy; this utilized a BAM file generated by BWA-MEM^35^ based on short-read sequencing data obtained in a prior genome survey^19^. Lastly, Purge Haplotigs^36^ was employed to identify and deduplicate haplotigs in the assembled genome.

**Table 1:** Sequencing data generated for *Pogonophryne albipinna* genome assembly and annotation.

Library type	Platform	Number of cells	Number of reads	Total read length (bp)
Long-reads	PacBio Sequel	6	7,776,779	81,108,670,479
Hi-C	Illumina Novaseq		733,064,394	110,692,723,494
Iso-seq	PacBio Sequel	2	37,596,041	62,649,769,489

### Hi-C sequencing and chromosome scaffolding

Muscle tissue was frozen and ground in liquid nitrogen for the construction of the Dovetail™ Hi-C library, following the instructions in the Dovetail™ Hi-C kit manual (Dovetail Genomics, Scotts Valley, CA, USA). Sequencing of the Hi-C library was performed on an Illumina NovaSeq. 6000 platform with a 2 × 150 bp paired-end run configuration. A total of 733,064,394 Hi-C reads, with an aggregate length of approximately 110.69 Gb (Table 1), were aligned to the draft genome assembly using Juicer v1.5.7^37^. Subsequently, a candidate assembly was produced using the 3D de novo assembly (3D-DNA) pipeline^38^. This candidate assembly underwent manual review, modification, and visualization via Juicebox v1.5^39^ to finalize both the genome assembly and the Hi-C contact map.

**Fig. 1:**
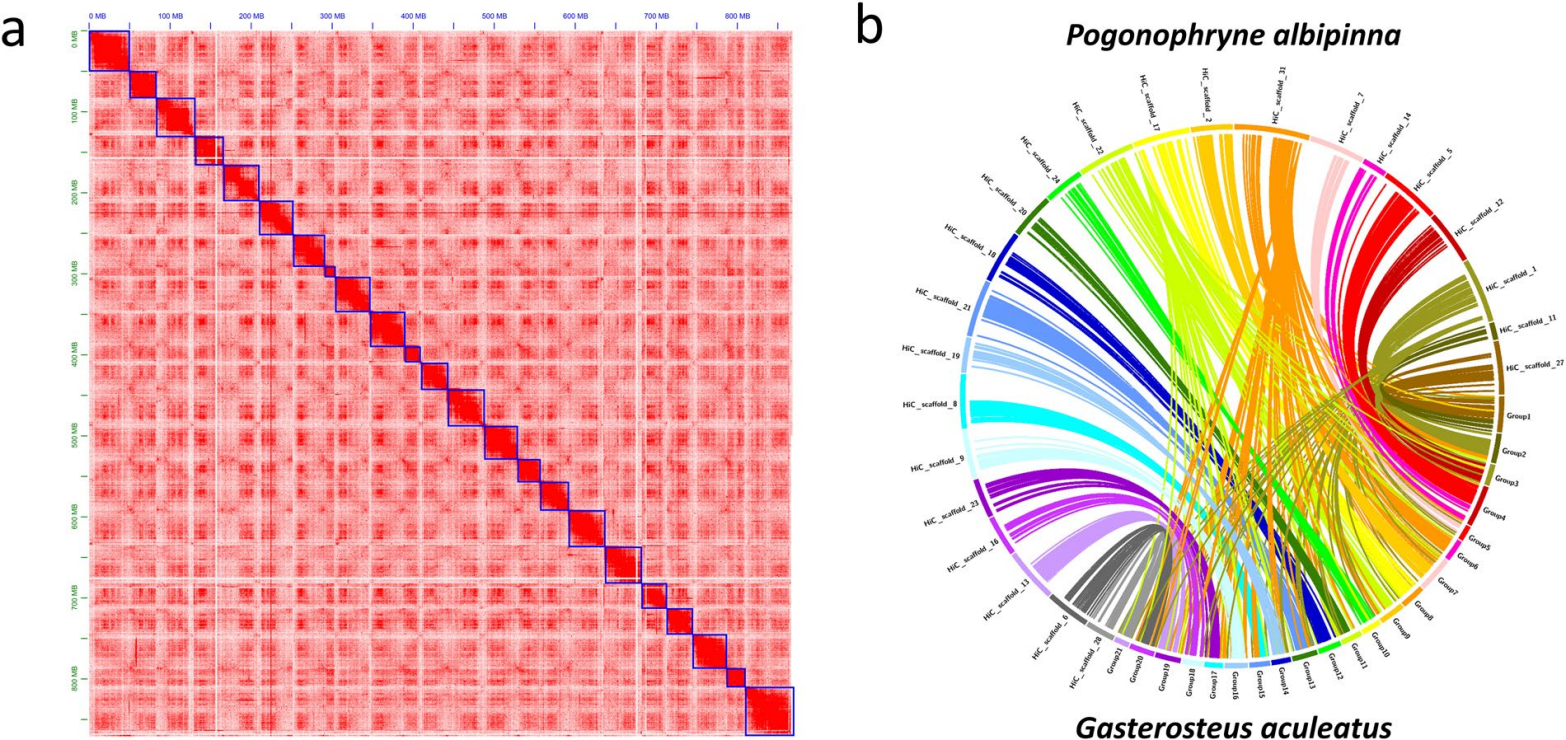
Chromosome-level genome assembly of *Pogonophryne albipinna*. (**a**) Hi-C interaction heat map for *P. albipinna*. The blue boxes represent the chromosomes. (**b**) Collinear relationship between *P. albipinna* and *Gasterosteus aculeatus*. Connections within the circle represent alignments between the two assemblies.

Our finalized genome assembly measured approximately 1.07 Gb with a maximum scaffold length of 59.39 Mb. We identified 1,111 Hi-C scaffolds, 23 of which exceeded 10 Mb in length, ranging between 13.61 Mb and 59.39 Mb (Table 2 and Table 3). These 23 pseudo-chromosomes in the *P. albipinna* genome aligned well with the 21 chromosomes of the *G. aculeatus* genome (Fig. 1). Notably, chromosomes from Group 1 and Group 4 of *G. aculeatus* corresponded to two chromosomes in *P. albipinna* each (HiC_scaffold_11 + 27 and HiC_scaffold_5 + 14). Karyotype studies have indicated that four out of the five species groups in the *Pogonophryne* genus possess 23 chromosome pairs^20,21^. This study was the first to identify these 23 scaffolds as chromosomes in *P. albipinna*, affirming that all groups within the *Pogonophryne* genus have a chromosomal count of 2n = 46.

**Table 2:** Statistics for *Pogonophryne albipinna* genome assembly.

Assembly	Hi-C
Number of scaffolds	1,111
Total size of scaffolds (bp)	1,074,502,020
Longest scaffold (bp)	59,391,674
N50 scaffold length (bp)	41,761,029
Number of scaffolds >10 Mb	23

**Table 3:** Lengths of *Pogonophryne albipinna* genome scaffolds (over 10 Mb).

No.	Scaffold name	Length (bp)
1	Chromosome_1	59,391,674
2	Chromosome_2	50,992,350
3	Chromosome_3	47,603,259
4	Chromosome_4	45,138,401
5	Chromosome_5	45,007,767
6	Chromosome_6	44,948,606
7	Chromosome_7	43,946,785
8	Chromosome_8	42,676,725
9	Chromosome_9	42,586,816
10	Chromosome_10	42,495,260
11	Chromosome_11	42,083,915
12	Chromosome_12	41,761,029
13	Chromosome_13	38,342,872
14	Chromosome_14	35,488,582
15	Chromosome_15	34,847,635
16	Chromosome_16	32,696,055
17	Chromosome_17	32,119,369
18	Chromosome_18	31,599,154
19	Chromosome_19	31,055,242
20	Chromosome_20	27,672,119
21	Chromosome_21	23,292,495
22	Chromosome_22	19,419,747
23	Chromosome_23	13,606,197

### Transcriptome sequencing

RNA was extracted from muscle tissue using the RNeasy Plus Mini kit (Qiagen, Hilden, Germany), in accordance with the manufacturer’s guidelines. Owing to the quality constraints of the RNA, different specimens were used for DNA and RNA isolation. For Iso-seq library construction, first-strand cDNA was synthesized using a SMARTer PCR cDNA synthesis kit (Clontech, Palo Alto, CA, USA). The SMRTbell library was then prepared as per the manufacturer’s protocol. Sequencing was conducted on a Sequel system (Pacific Biosciences, Menlo Park, CA, USA) using two SMRT cells 1 M v3 LR and Sequel sequencing chemistry 3.0. Iso-seq produced 37,596,041 subreads with a total of 62.65 Gb of nucleotides (Table 1). Analysis of Iso-seq data was performed using the Iso-seq 3 pipeline in SMRT Link v6.0.0 with default settings.

### Repeat analysis and masking

**Fig. 2:**
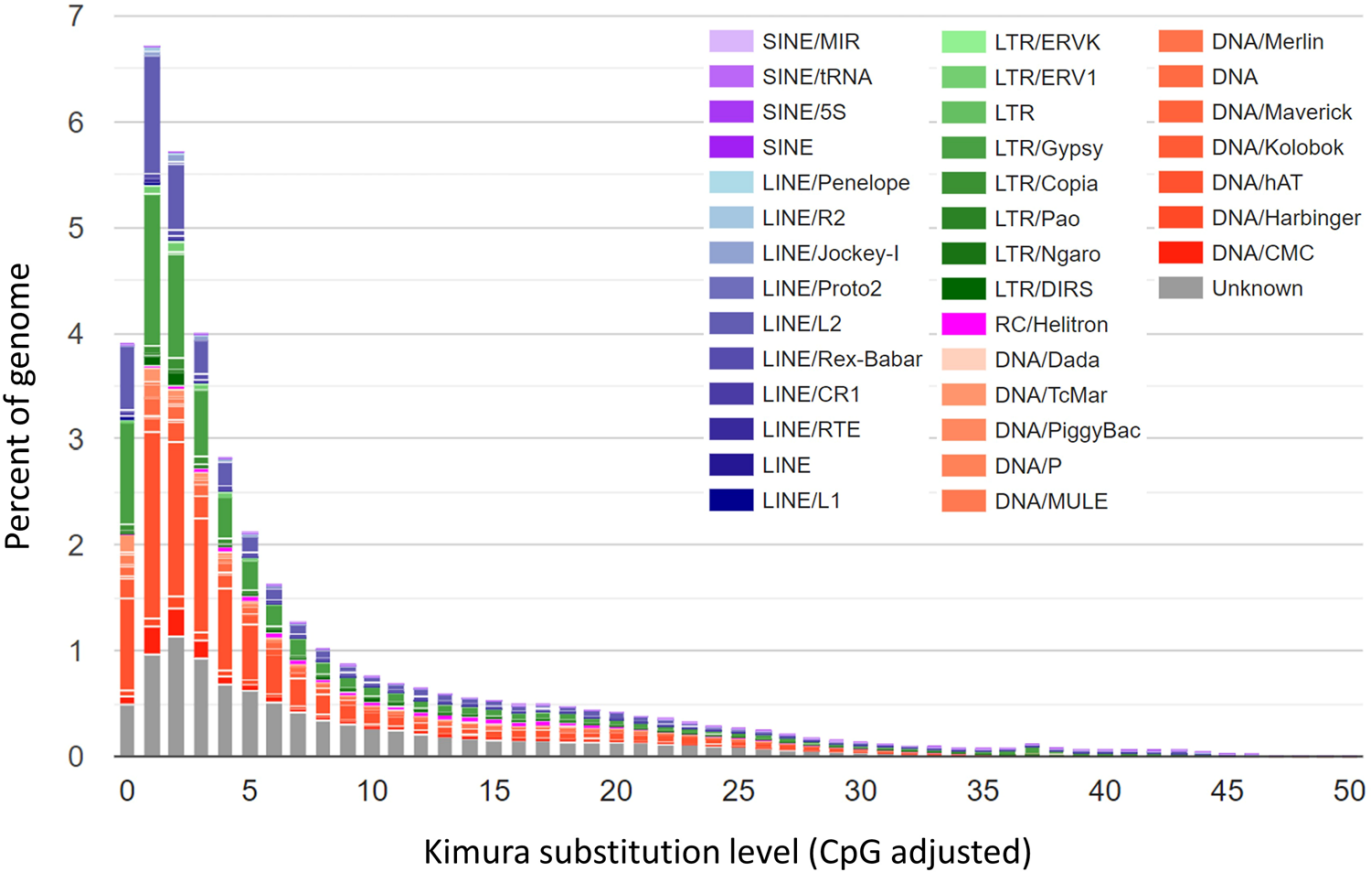
Kimura distance-based copy divergence analysis of transposable elements in teleost genomes. The graphs depict genome coverage (Y-axis) for each type of TE in the *Pogonophryne albipinna* genome.

A de novo repeat library was generated using RepeatModeler v1.0.3^40^, incorporating the utilities RECON v1.08^41^, RepeatScout v1.0.5^42^ and Tandem Repeats Finder v4.09^43^, all of which operated with default parameters. All repeats identified by RepeatModeler, except for transposons, were cross-referenced with the UniProt/SwissProt database^44^. To specifically identify long terminal repeat retrotransposons (LTR-RTs), LTR_retriever was executed^45^, utilizing raw LTR data sourced from LTRharvest^46^ and LTR_FINDER^47^. The assembled repeat library was then utilized to mask repetitive elements via RepeatMasker v4.0.9, accessed on November 24, 2020, from https://www.repeatmasker.org/. Analysis revealed that the *P. albipinna* genome comprises 50.68% repetitive sequences, of which 48.03% were transposable elements (TEs), including short interspersed nuclear elements (SINEs, 0.29%), long interspersed nuclear elements (LINEs, 5.50%), long terminal repeats (LTRs, 17.91%), and DNA transposons (15.38%) (Table 4). Kimura divergence values for each alignment were calculated, and the interspersed repeat landscape was plotted using the scripts “calcDivergenceFromAlign.pl” and “createRepeatLandscape.pl”. The Kimura distances for all TE copies indicated that the *P. albipinna* genome harbored a greater number of recent TE copies with Kimura divergence K-values ≤ 5, primarily influenced by Gypsy LTR and hAT DNA elements (Fig. 2).

**Table 4:** Statistics for annotated *Pogonophryne albipinna* transposable elements.

Class	Number of elements	Length occupied (bp)	Percentage of sequence (%)
SINEs:	20,523	3,063,718	0.29%
MIRs	13,231	2,031,457	0.19%
LINEs:	174,171	59,316,996	5.50%
LINE1	4,887	1,339,549	0.12%
LINE2	110,647	43,014,081	4.00%
LTR elements:	471,673	192,373,177	17.91%
Gypsy	138,972	60,560,082	5.64%
DIRSs	10,542	6,693,107	0.62%
RC: Helitrons	10,174	5,687,850	0.53%
DNA elements:	531,943	165,424,411	15.38%
hAT-Ac	141,912	43,112,186	4.01%
Unclassified:	431,689	90,165,374	8.39%
Total interspersed repeats:	1,640,173	516,031,526	48.03%
Low complexity:	24,782	1,477,563	0.14%
Satellites:	11,185	2,123,109	0.20%
Simple repeats:	277,977	24,329,247	2.26%
Ribosomal RNAs:	286	30,590	0.00%
Small nuclear RNAs:	511	169,931	0.02%
Transfer RNAs:	1,822	389,123	0.04%
Total bases masked:	1,956,736	544,551,089	50.68%

### Gene prediction and functional annotation

**Fig. 3:**
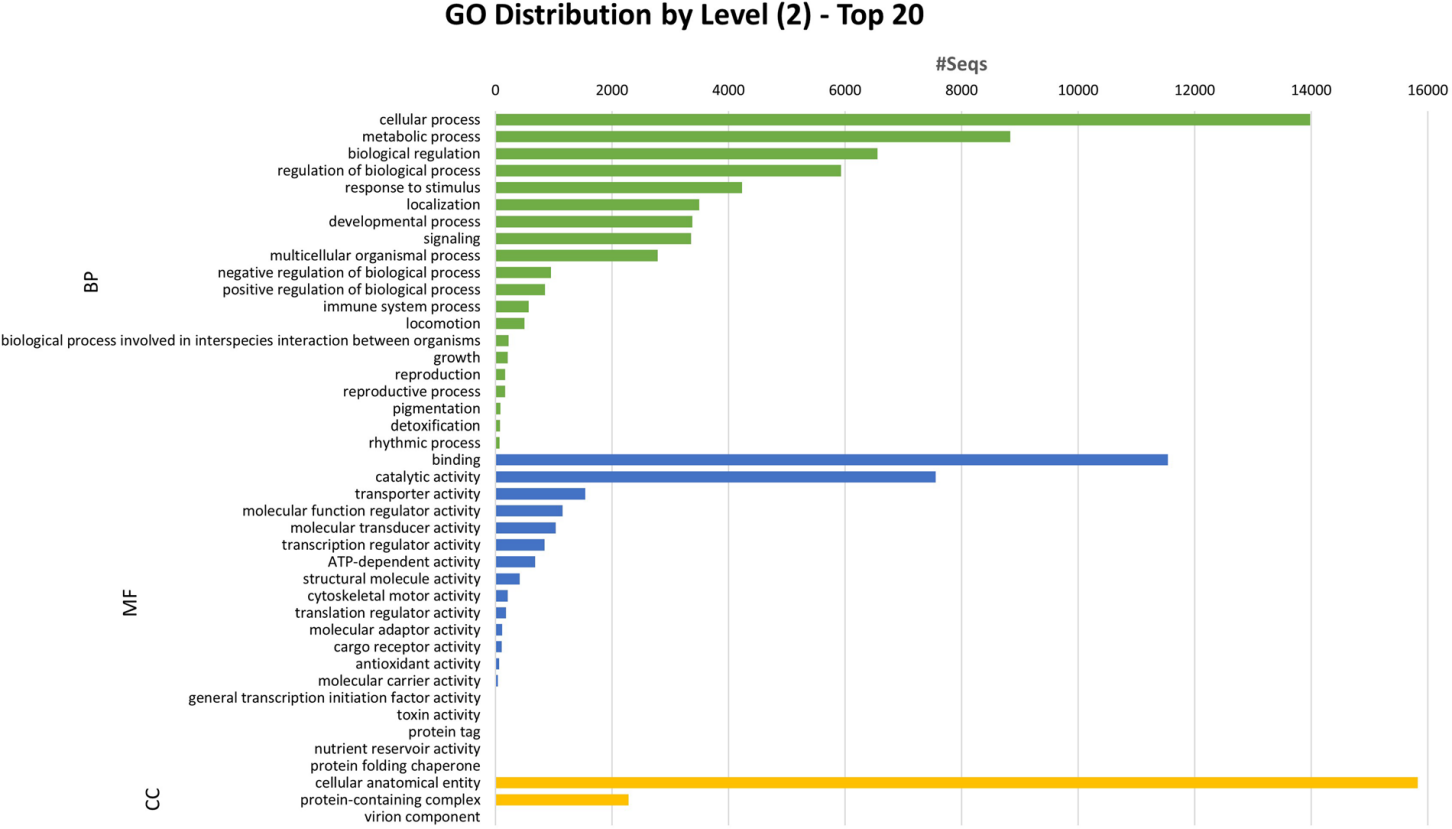
Gene ontology (GO) annotations of the predicted genes in the *Pogonophryne albipinna* genome. The horizontal axis indicates the number of genes in each class, while the vertical axis indicates the classes in the 2-level GO-annotation.

Gene structure annotation was conducted using EVidenceModeler (EVM) v1.1.1^48^, integrating multiple types of evidence for gene prediction. Initially, the Program to Assemble Spliced Alignments (PASA) pipeline v2.5.1^49^ was applied to Iso-seq data to generate transcript evidence. Ab initio gene prediction on the repeat-masked genome assembly was then performed using GeneMark-ES v4.68^50^. Protein hints were generated using Actinopterygii protein sequences from the SwissProt database^44^ using ProtHint v2.6.0^51^. These hints were employed to produce protein-based evidence via GeneMark-EP+ v4.68^51^ and for ab initio gene prediction with Augustus v3.4.0^52^. EVM combined all gene models, assigning weight values to each type of evidence (ABINITIO_PREDICTION, 1; PROTEIN, 50; TRANSCRIPT, 50) to produce a consensus gene structure. The consensus gene prediction was further refined using the PASA pipeline^49^ to include untranslated regions (UTRs) and alternatively spliced isoforms, based on Iso-seq data. In the *P. albipinna* genome assembly, EVM pipeline predicted a total of 31,128 protein-coding genes (Table 5). The cumulative lengths of exons and coding sequences were 48.20 Mb and 43.33 Mb, respectively, averaging 8.46 exons per gene (Table 5). Functional annotation of the predicted genes was performed by aligning them to the NCBI non-redundant protein (nr) database^53^ using BLASTP v2.9.0^54^, with an e-value cutoff set at 1e-5. Protein functions were predicted using InterProScan v5.44.79^55^ on the translated protein sequences from the transcripts. Gene Ontology (GO) terms were assigned to the sequences using the Blast2GO^56^ module in OmicsBox v1.3.11^57^. Kyoto Encyclopedia of Genes and Genomes (KEGG) pathway annotation was accomplished using the KEGG Automatic Annotation Server (KAAS)^58^ and KEGG Mapper^59^. Trinotate v3.2.0^60^ provided a comprehensive functional annotation of the transcriptome sequences. Specifically, coding regions were identified using TransDecoder v5.5.0, followed by sequence homology searches using BLAST^54^ against the UniProt/SwissProt database^44^. Protein domain identification was performed using HMMER^61^ via the Pfam database^62^, while protein signal peptides were predicted with SignalP v5.0^63^ and transmembrane domains with TMHMM v2.0^64^. Consequently, 30,992 genes (99.56%) were annotated in at least one database (Table 5). Among these, 26,292 genes (84.5%) received annotations in the GO database (Table 5), and the distribution of GO terms is presented in Fig. 3.

**Table 5:** Statistics for *Pogonophryne albipinna* genome annotation.

	Count	Length Sum (bp)
Annotation database	Annotated number	Percentage (%)
Exon	263,211	48,199,293
CDS	261,649	43,329,592
No. Genes	31,128	
nr	30,784	98.9
GO	26,292	84.5
KEGG	15,939	51.2
SwissProt blastx	25,041	80.4
SwissProt blastp	24,616	79.1
Pfam	22,314	71.7
SignalP	28,617	91.9
TmHMM	8,504	27.3
InterProScan	29,121	93.6

### Gene family identification and phylogenetic analysis

**Fig. 4:**
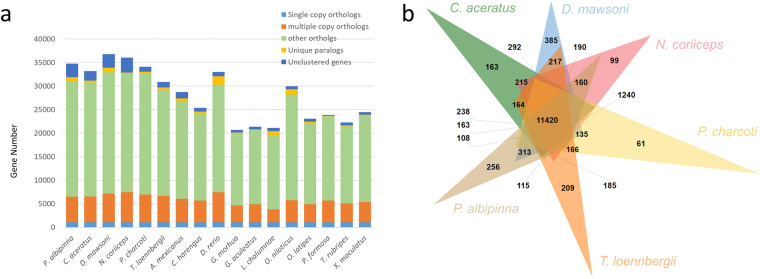
Gene family comparison. (**a**) Orthologous gene families between *Pogonophryne albipinna* and other fish species. (**b**) Venn diagram showing orthologous gene families among *P. albipinna* and five other Antarctic fish species.

**Fig. 5:**
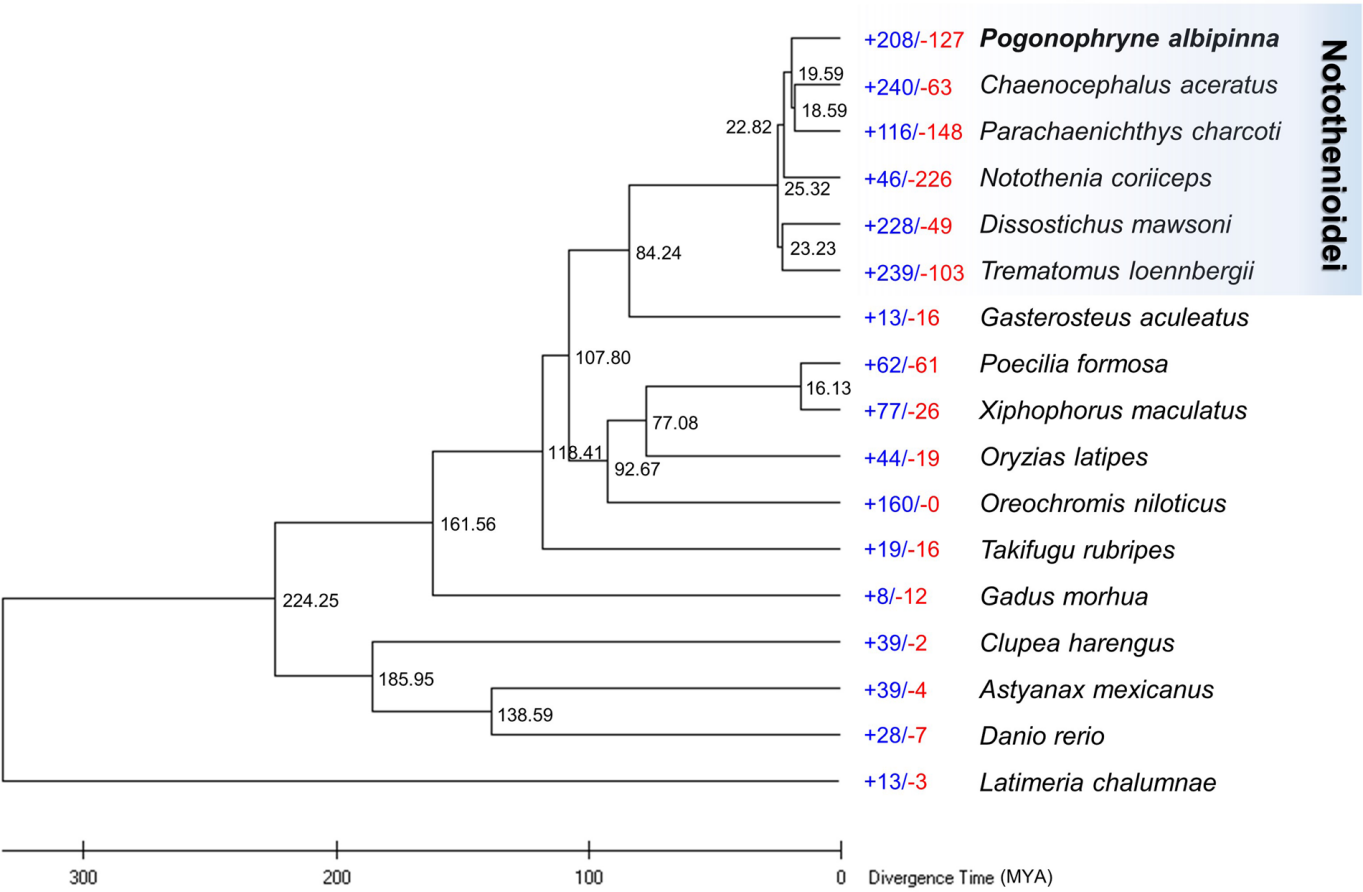
Phylogenetic analysis of *Pogonophryne albipinna* within the teleost lineage and analysis of gene family gains and losses, including the number of gained gene families (+) and lost gene families (−). Each branch site number indicates divergence times between lineages.

Protein sequences from sixteen teleost species were obtained, with only the longest transcript variant of each gene being selected for further analysis (Table S1). Orthogroups for 17 teleost species were determined based on protein sequence similarity using OrthoFinder v2.4.0^65^ with default parameters. The analysis revealed that 6,727 orthogroups were shared across all 17 species, while 186 orthogroups, encompassing 766 genes, were specific to *P. albipinna* (Fig. 4a, Table S2). A maximum-likelihood (ML) phylogenetic tree was constructed using the concatenated protein sequences of 1,092 single-copy orthologous genes common to the 17 teleost species, employing MEGA X software^66^. Divergence times were estimated using TimeTree^67^, with median estimates for *Gadus morhua* and *Danio rerio* set at 224 million years ago. In the resulting tree, *P. albipinna* clustered with five other Antarctic fish species, diverging from a common ancestor with *G. aculeatus* approximately 84.24 million years ago (Fig. 5). The divergence time between *P. albipinna* and *N. coriiceps* was estimated to be around 22.82 million years ago, followed by a separation from the *C. aceratus*/*P. charcoti* clade about 19.59 million years ago (Fig. 5). Gene family expansions and contractions were analyzed using CAFE v4.2.1^68^, with the parameters -p 0.05 and -filter. The analysis revealed that the *P. albipinna* genome had 208 significantly expanded and 127 significantly contracted gene families (Fig. 5). Expanded gene families in *P. albipinna* were enriched in telomere-related biological process GO terms (Table S3). GO enrichment analysis results for genes in expanded, contracted, and *P. albipinna*-specific gene families are presented in Tables S3–5. Comparative analysis of orthologous gene clusters among six Antarctic fish species (*P. albipinna, C. aceratus, D. mawsoni, N. coriiceps, P. charcoti*, and *T. loennbergii*) was conducted and visualized using OrthoVenn3^69^. In these analyses, 11,420 orthologous gene families were commonly identified among the six Antarctic species, while 256 gene families were unique to the *P. albipinna* genome (Fig. 4b).

## Data Records

The final genome assembly of *Pogonophryne albipinna* has been deposited in GenBank with the accession number JAPTMU000000000^70^. The PacBio (SRR26989350), Hi-C (SRR26989351), and Iso-seq (SRR26989352) reads have been deposited in the NCBI Sequence Read Archive (SRA) database under study accession number of SRP304454^71^.

## Technical Validation

### Quality control of nucleic acids and libraries

The quality and quantity of the extracted DNA were assessed using a Qubit 2.0 fluorometer (Invitrogen, Life Technologies, Carlsbad, CA, USA) and a Fragment Analyzer (Agilent Technologies, Santa Clara, CA, USA). The main peak of the input genomic DNA was 28 kb and the final size of the SMRTbell library for long-read sequencing was ~24 kb. The size distribution of Hi-C fragments was centered around 200 bp and the final size-selected Hi-C library was distributed a size range of 200 bp to 1 kb. The RNA quality and quantity were assessed using a 2100 Bioanalyzer (Agilent Technologies, Santa Clara, CA, USA) and a Qubit 2.0 fluorometer (Invitrogen, Life Technologies, CA, USA), respectively. The RNA integrity number (RIN) value of the total RNA was 8.8 and the average library size for Iso-seq was ~2,800 bp.

### Evaluation of genome assembly and annotation

To evaluate the assembly’s completeness, we used Benchmarking Universal Single-Copy Orthologs (BUSCO) v4.1.2^72^ in genome assessment mode, employing the Actinopterygii_odb10 dataset. The assembly showed 95.6% (3,479) complete and 1.2% (42) fragmented genes among 3,640 Actinopterygii single-copy orthologs (Table 6). Additionally, BUSCO v4.1.2^72^ in transcriptome assessment mode represented 85.4% (3,109) of completed and 3.1% (112) of fragmented BUSCOs in actinopterygii_odb10 dataset. The assembly’s contiguity was assessed using the N50 value, defined as the length of the shortest contig or scaffold constituting 50% of the total genome length. The N50 value for the *P. albipinna* genome assembly was 41.76 Mb (Table 2). Quality value (QV) and k-mer completeness were estimated using Merqury v1.3^73^, resulting in a QV of 39.15 and completeness of 93.48% (Table 7). These metrics indicate high base-level accuracy and completeness for the assembly.

**Table 6:** Completeness of the *Pogonophryne albipinna* genome assembly and annotation evaluated with Benchmarking Universal Single-Copy Orthologs (BUSCO).

Actinopterygii_odb10	Genome		Transcriptome	
	Number	Percentage (%)	Number	Percentage (%)
Complete BUSCOs (C)	3,479	95.6	3,109	85.4
Complete and single-copy BUSCOs (S)	3,407	93.6	2,924	80.3
Complete and duplicated BUSCOs (D)	72	2.0	185	5.1
Fragmented BUSCOs (F)	42	1.2	112	3.1
Missing BUSCOs (M)	119	3.2	419	11.5
Total BUSCO groups searched	3,640		3,640

**Table 7:** Assembly validation of *Pogonophryne albipinna* genome using Merqury.

Quality value (QV)	k-mer error rate	k-mer completeness (%)
39.15	1.22E-04	93.48

### Supplementary information

Supplementary Information

## Data Availability

All bioinformatic software and pipeline used in this study were implemented according to the protocols provided by the software developers. The versions and parameters for each software can be found in the Methods section. Unless otherwise stated, default parameters were employed.
