# Supplementary material for: Chromosome-level genome assembly and annotation of the Antarctica whitefin plunderfish Pogonophryne albipinna
Source: Sci Data. 2023 Dec 12;10:891. doi: 10.1038/s41597-023-02811-x (PMC10716251; doi:10.1038/s41597-023-02811-x)
Supplement: Supplementary file 1 — Supplementary Information [file 41597_2023_2811_MOESM1_ESM.pdf]

Supplementary Information

Chromosome-level genome assembly and annotation of the whitefin plunderfish *Pogonophryne albipinna*

Euna Jo[1,2,†], Soyun Choi[1,†], Seung Jae Lee[1], Jinmu Kim[1], Eun Kyung Choi[1], Minjoo Cho[1], Jangyeon Kim[1], Sangdeok Chung[3], Jaebong Lee[3], Jeong-Hoon Kim[2] and Hyun Park[1,*]

[1] Department of Biotechnology, College of Life Sciences and Biotechnology, Korea University, Seoul 02841, Korea
[2] Division of Life Sciences, Korea Polar Research Institute (KOPRI), Incheon 21990, Korea
[3] National Institute of Fisheries Science (NIFS), Busan, 46083 Korea

[†]These authors contributed equally to this work.
[*]Corresponding author: Hyun Park (hpark@korea.ac.kr)

Table of Contents

Table S1. Species information used in the orthologous gene family analysis.
Table S2. Summary of gene families of *Pogonophryne albipinna* and other fish species.
Table S3. Gene Ontology of expanded gene families in the *Pogonophryne albipinna* genome among 17 fish species.
Table S4. Gene Ontology of contracted gene families in the *Pogonophryne albipinna* genome among 17 fish species.
Table S5. Gene Ontology of *Pogonophryne albipinna*-specific gene families among six Antarctic fish species.

Table S1. Species information used in the orthologous gene family analysis.

Common name	Species	Assembly
Whitefin plunderfish	*Pogonophryne albipinna*	This study
Pachon cavefish	*Astyanax mexicanus*	Astyanax_mexicanus-1.0.2
Blackfin icefish	*Chaenocephalus aceratus*	Kim et al., 2019*
Atlantic herring	*Clupea harengus*	Ch_v2.0.2
Zebrafish	*Danio rerio*	GRCz11
Antarctic toothfish	*Dissostichus mawsoni*	GCA_011823955.1
Atlantic cod	*Gadus morhua*	gadMor1
Stickleback	*Gasterosteus aculeatus*	BROAD S1
Coelacanth	*Latimeria chalumnae*	LatCha1
Antarctic bullhead notothen	*Notothenia coriiceps*	GCA_000735185.1
Nile tilapia	*Oreochromis niloticus*	O_niloticus_UMD_NMBU
Japanese medaka HNI	*Oryzias latipes*	ASM223471v1
Antarctic dragonfish	*Parachaenichthys charcoti*	Ahn et al., 2017**
Amazon molly	*Poecilia formosa*	Poecilia_formosa-5.1.2
Fugu	*Takifugu rubripes*	fTakRub1.2
Antarctic scaly rockcod	*Trematomus loennbergii*	GCA_012861695.1
Platyfish	*Xiphophorus maculatus*	X_maculatus-5.0-male

* Kim et al., 2019, Nature Ecology & Evolution, 3, 469–478.

** Ahn et al., 2017, GigaScience, 6, gix060.

Table S2. Summary of gene families of *Pogonophryne albipinna* and other fish species.

	Number of genes	Number of genes in orthogroups	Number of unassigned genes	Percentage of genes in orthogroups	Percentage of unassigned genes	Number of orthogroups containing species	Percentage of orthogroups containing species	Number of species-specific orthogroups	Number of genes in species-specific orthogroups	Percentage of genes in species-specific orthogroups
P. albipinna	31,128	28,247	2,881	90.7	9.3	15,920	63.1	186	766	2.5
C. aceratus	30,773	28,780	1,993	93.5	6.5	17,112	67.9	136	399	1.3
D. mawsoni	32,914	30,055	2,859	91.3	8.7	16,907	67.1	320	996	3.0
N. coriiceps	32,661	29,475	3,186	90.2	9.8	18,185	72.1	86	205	0.6
P. charcoti	32,713	31,686	1,027	96.9	3.1	18,458	73.2	72	368	1.1
T. loennbergii	29,163	28,012	1,151	96.1	3.9	15,164	60.1	159	537	1.8
A. mexicanus	26,698	25,361	1,337	95.0	5.0	15,214	60.3	149	728	2.7
C. harengus	24,095	23,341	754	96.9	3.1	14,156	56.1	119	512	2.1
D. rerio	30,313	29,448	865	97.1	2.9	14,906	59.1	213	1,803	5.9
G. morhua	20,095	19,518	577	97.1	2.9	13,679	54.3	16	40	0.2
G. aculeatus	20,787	20,231	556	97.3	2.7	13,879	55.0	15	44	0.2
L. chalumnae	19,569	18,923	646	96.7	3.3	13,090	51.9	126	887	4.5
O. niloticus	28,189	27,607	582	97.9	2.1	15,056	59.7	143	1,157	4.1
O. latipes	22,127	21,483	644	97.1	2.9	14,239	56.5	41	311	1.4
P. formosa	23,615	23,443	172	99.3	0.7	14,924	59.2	25	75	0.3
T. rubripes	21,411	20,789	622	97.1	2.9	13,833	54.9	58	258	1.2
X. maculatus	23,774	23,258	516	97.8	2.2	14,993	59.5	36	159	0.7

Table S3. Gene Ontology of expanded gene families in the *Pogonophryne albipinna* genome among 17 fish species.

Category	GO ID	GO name	No. genes	P-value
Biological process	GO:0090305	nucleic acid phosphodiester bond hydrolysis	88	7.90E-23
	GO:0032212	positive regulation of telomere maintenance via telomerase	16	3.18E-15
	GO:0051781	positive regulation of cell division	27	4.38E-15
	GO:0051673	perturbation of plasma membrane integrity in another organism	16	1.84E-14
	GO:0050830	defense response to Gram-positive bacterium	16	1.08E-12
	GO:0006888	endoplasmic reticulum to Golgi vesicle-mediated transport	30	8.89E-12
	GO:1904908	negative regulation of maintenance of mitotic sister chromatid cohesion, telomeric	10	4.23E-11
	GO:0051973	positive regulation of telomerase activity	10	4.23E-11
	GO:1904355	positive regulation of telomere capping	10	4.23E-11
	GO:0070198	protein localization to chromosome, telomeric region	10	4.23E-11
	GO:0070212	protein poly-ADP-ribosylation	10	4.23E-11
	GO:0070213	protein auto-ADP-ribosylation	10	4.23E-11
	GO:1904743	negative regulation of telomeric DNA binding	10	4.23E-11
	GO:1904357	negative regulation of telomere maintenance via telomere lengthening	10	4.27E-10
	GO:0006897	endocytosis	54	1.98E-09
	GO:0051225	spindle assembly	16	1.84E-08
	GO:0018107	peptidyl-threonine phosphorylation	10	2.99E-08
	GO:2000178	negative regulation of neural precursor cell proliferation	8	4.17E-08
	GO:0035821	modulation of process of another organism	7	1.67E-06
	GO:0042593	glucose homeostasis	14	1.78E-05
	GO:0018105	peptidyl-serine phosphorylation	10	3.78E-05
	GO:0006334	nucleosome assembly	11	4.04E-05
	GO:0045087	innate immune response	24	5.09E-05
	GO:0021628	olfactory nerve formation	4	7.12E-05
	GO:0090263	positive regulation of canonical Wnt signaling pathway	10	8.22E-05
	GO:0032508	DNA duplex unwinding	19	9.06E-05
	GO:0044179	hemolysis in another organism	7	2.97E-04
	GO:0071699	olfactory placode morphogenesis	4	3.30E-04
	GO:0007052	mitotic spindle organization	10	4.10E-04
	GO:0015671	oxygen transport	7	4.66E-04

	GO:0043066	negative regulation of apoptotic process	18	7.16E-04
	GO:1902340	negative regulation of chromosome condensation	3	7.75E-04
	GO:0090230	regulation of centromere complex assembly	3	7.75E-04
	GO:0007566	embryo implantation	3	7.75E-04
	GO:0048484	enteric nervous system development	8	1.73E-03
	GO:0030901	midbrain development	8	1.73E-03
	GO:1900748	positive regulation of vascular endothelial growth factor signaling pathway	4	1.98E-03
	GO:0031509	subtelomeric heterochromatin formation	3	2.89E-03
	GO:0031508	pericentric heterochromatin formation	3	2.89E-03
	GO:0007140	male meiotic nuclear division	3	2.89E-03
	GO:0070374	positive regulation of ERK1 and ERK2 cascade	4	6.14E-03
	GO:0016024	CDP-diacylglycerol biosynthetic process	5	6.44E-03
	GO:0017038	protein import	3	6.72E-03
	GO:0000209	protein polyubiquitination	10	6.74E-03
	GO:0008584	male gonad development	3	6.72E-03
	GO:0006308	DNA catabolic process	5	8.93E-03
	GO:0090090	negative regulation of canonical Wnt signaling pathway	8	1.24E-02
	GO:0007156	homophilic cell adhesion via plasma membrane adhesion molecules	23	1.96E-02
	GO:0009253	peptidoglycan catabolic process	3	2.04E-02
	GO:0007286	spermatid development	3	2.04E-02
	GO:0035264	multicellular organism growth	3	4.27E-02
	GO:0051028	mRNA transport	9	4.11E-02
	GO:0045893	positive regulation of DNA-templated transcription	29	3.09E-02
	GO:0051492	regulation of stress fiber assembly	4	2.59E-02
Molecular function	GO:0097020	COPII receptor activity	30	1.95E-30
	GO:0005200	structural constituent of cytoskeleton	35	2.21E-21
	GO:0042393	histone binding	28	4.84E-15
	GO:0002039	p53 binding	18	1.63E-14
	GO:0016712	oxidoreductase activity, acting on paired donors, with incorporation or reduction of molecular oxygen, reduced flavin or flavoprotein as one donor, and incorporation of one atom of oxygen	23	3.90E-13
	GO:0020037	heme binding	44	4.92E-12
	GO:0046982	protein heterodimerization activity	38	5.86E-12

	GO:0004499	N,N-dimethylaniline monooxygenase activity	11	4.26E-11
	GO:0106294	NADPH oxidase H202-forming activity	11	4.26E-11
	GO:0008201	heparin binding	27	2.90E-10
	GO:1990404	NAD+-protein ADP-ribosyltransferase activity	10	9.33E-09
	GO:0005044	scavenger receptor activity	24	1.20E-08
	GO:0008013	beta-catenin binding	18	6.94E-08
	GO:0003950	NAD+ ADP-ribosyltransferase activity	16	1.19E-07
	GO:0090729	toxin activity	7	4.04E-07
	GO:0005506	iron ion binding	36	5.74E-07
	GO:0004803	transposase activity	9	2.90E-06
	GO:0008821	crossover junction DNA endonuclease activity	5	6.53E-06
	GO:0140658	ATP-dependent chromatin remodeler activity	18	7.33E-06
	GO:0050661	NADP binding	12	1.38E-05
	GO:0003964	RNA-directed DNA polymerase activity	8	3.29E-05
	GO:0003678	DNA helicase activity	19	6.27E-05
	GO:0048306	calcium-dependent protein binding	7	1.82E-04
	GO:0000979	RNA polymerase II core promoter sequence-specific DNA binding	4	3.30E-04
	GO:0005344	oxygen carrier activity	7	4.66E-04
	GO:0019825	oxygen binding	7	4.66E-04
	GO:0008083	growth factor activity	27	4.77E-04
	GO:0098770	FBXO family protein binding	3	7.75E-04
	GO:0005525	GTP binding	72	7.61E-04
	GO:0030527	structural constituent of chromatin	3	7.75E-04
	GO:0005219	ryanodine-sensitive calcium-release channel activity	5	1.88E-03
	GO:0008146	sulfotransferase activity	15	2.33E-03
	GO:0031492	nucleosomal DNA binding	4	3.68E-03
	GO:0004605	phosphatidate cytidylyltransferase activity	5	4.47E-03
	GO:0003796	lysozyme activity	3	6.72E-03
	GO:0004634	phosphopyruvate hydratase activity	4	1.38E-02
Cellular component	GO:0000786	nucleosome	38	1.73E-22
	GO:0000242	pericentriolar material	10	4.23E-11
	GO:0071339	MLL1 complex	18	5.56E-11
	GO:0097431	mitotic spindle pole	10	4.27E-10
	GO:0000781	chromosome, telomeric region	13	8.65E-07
	GO:0016529	sarcoplasmic reticulum	14	6.87E-06
	GO:0005833	hemoglobin complex	7	5.72E-05
	GO:0005874	microtubule	38	2.83E-04
	GO:0000791	euchromatin	4	3.30E-04
	GO:0001740	Barr body	3	7.75E-04
	GO:0005700	polytene chromosome	3	7.75E-04
	GO:0000939	inner kinetochore	3	6.72E-03
	GO:0005643	nuclear pore	10	6.74E-03

	GO:0070062	extracellular exosome	3	1.25E-02
	GO:0000015	phosphopyruvate hydratase complex	4	1.38E-02
	GO:0005637	nuclear inner membrane	4	3.36E-02

Table S4. Gene Ontology of contracted gene families in the *Pogonophryne albipinna* genome among 17 fish species.

Category	GO ID	GO name	No. genes	P-value
Biological process	GO:0050911	detection of chemical stimulus involved in sensory perception of smell	2	3.01E-04
	GO:0030259	lipid glycosylation	1	3.02E-02
	GO:0051209	release of sequestered calcium ion into cytosol	2	4.11E-03
	GO:0009452	7-methylguanosine RNA capping	1	3.02E-02
Molecular function	GO:0015020	glucuronosyltransferase activity	3	5.05E-05
	GO:0004984	olfactory receptor activity	2	3.01E-04
	GO:0005219	ryanodine-sensitive calcium-release channel activity	2	7.80E-04
	GO:0030246	carbohydrate binding	3	2.33E-02
	GO:0001594	trace-amine receptor activity	1	3.82E-03
	GO:0005516	calmodulin binding	3	6.74E-03
	GO:0005540	hyaluronic acid binding	2	4.11E-03
	GO:0008307	structural constituent of muscle	1	1.14E-02
Cellular component	GO:0033017	sarcoplasmic reticulum membrane	3	1.04E-04
	GO:0034993	meiotic nuclear membrane microtubule tethering complex	2	3.01E-04
	GO:0001527	microfibril	1	1.14E-02
	GO:0005640	nuclear outer membrane	1	2.65E-02
	GO:0005923	bicellular tight junction	2	2.36E-02

Table S5. Gene Ontology of *Pogonophryne albipinna*-specific gene families among six Antarctic fish species.

Category	GO ID	GO name	No. genes	P-value
Biological process	GO:0006313	DNA transposition	117	1.22E-70
	GO:0015074	DNA integration	112	6.15E-63
	GO:0006278	RNA-templated DNA biosynthetic process	6	1.08E-03
	GO:0060134	prepulse inhibition	1	3.41E-02
	GO:0050830	defense response to Gram-positive bacterium	4	6.04E-03
	GO:0090305	nucleic acid phosphodiester bond hydrolysis	20	4.51E-03
	GO:1901537	positive regulation of DNA demethylation	1	3.41E-02
	GO:0034316	negative regulation of Arp2/3 complex-mediated actin nucleation	1	3.41E-02
	GO:0034080	CENP-A containing chromatin assembly	1	3.41E-02
	GO:0050911	detection of chemical stimulus involved in sensory perception of smell	2	2.18E-02
	GO:0106300	protein-DNA covalent cross-linking repair	1	3.41E-02
	GO:0034154	toll-like receptor 7 signaling pathway	1	3.41E-02
	GO:0051673	perturbation of plasma membrane integrity in another organism	4	3.47E-03
	GO:0021942	radial glia guided migration of Purkinje cell	1	3.41E-02
	GO:0070537	histone H2A K63-linked deubiquitination	1	3.41E-02
Molecular function	GO:0003677	DNA binding	146	2.24E-15
	GO:0003964	RNA-directed DNA polymerase activity	6	9.31E-06
	GO:0035615	clathrin adaptor activity	5	1.46E-04
	GO:0004803	transposase activity	5	1.46E-04
	GO:0017128	phospholipid scramblase activity	2	1.59E-02
	GO:0031386	protein tag	2	1.59E-02
	GO:0004518	nuclease activity	20	1.17E-02
	GO:0004984	olfactory receptor activity	2	2.18E-02
	GO:0004965	G protein-coupled GABA receptor activity	2	4.37E-02
	GO:0002020	protease binding	2	2.84E-02
	GO:0008273	calcium, potassium:sodium antiporter activity	2	1.59E-02
	GO:0000062	fatty-acyl-CoA binding	3	1.09E-02
	GO:0046982	protein heterodimerization activity	11	2.92E-03
Cellular component	GO:0000786	nucleosome	13	3.64E-07
	GO:0030121	AP-1 adaptor complex	5	2.98E-05
	GO:0031315	extrinsic component of mitochondrial outer membrane	2	1.16E-03
	GO:1902711	GABA-A receptor complex	2	3.57E-02

	GO:0009706	chloroplast inner membrane	1	3.41E-02
	GO:1990221	L-cysteine desulfurase complex	1	3.41E-02
	GO:0005905	clathrin-coated pit	5	1.24E-02
	GO:0031105	septin complex	2	1.59E-02
	GO:0036157	outer dynein arm	1	3.41E-02
